# Patient-Reported Outcomes for Acute Gallstone Pathology

**DOI:** 10.1007/s00268-016-3854-x

**Published:** 2017-01-10

**Authors:** Ed Parkin, Martyn Stott, Joy Brockbank, Simon Galloway, Ian Welch, Andrew Macdonald

**Affiliations:** 1 Department of Upper Gastrointestinal Surgery, University Hospital of South Manchester, Manchester, UK; 2 Obesity and Cancer Research Group, Institute of Cancer Sciences, University of Manchester, Manchester Academic Health Science Centre, The Christie NHS Foundation Trust, Wilmslow Road, Manchester, M20 4BX UK

## Abstract

**Background:**

A number of prominent surgical trials and clinical guidelines regard length of hospital stay and rates of daycase surgery as being of upmost importance following cholecystectomy. However, it is unclear whether these outcomes also matter to patients. This study aimed to identify the factors patients regard as most important when admitted with acute gallstone pathology.

**Methods:**

A 41-item survey was produced by combining outcomes assessed in recent clinical trials with results from a preliminary patient questionnaire. This was then given out prospectively to patients presenting with acute gallstone pathology, prior to their cholecystectomy. Patients were asked to read an information sheet about laparoscopic cholecystectomy and then complete the survey, scoring each item out of 100 in terms of importance to them.

**Results:**

Fifty-six patients completed the survey (43 females; median age 51 years). Diagnoses were: cholecystitis (28 patients), biliary colic (13), pancreatitis (10), common bile duct stones (3) and cholangitis (2). The top-scoring survey item was “long-term quality of life after surgery”, with a median value of 97 out of 100. Other high-scoring items included “cleanliness of the ward environment” and “pain control after surgery” (both 96). The lowest-scoring item was “being treated as a daycase” (54).

**Conclusion:**

Patients with acute gallstone pathology view long-term quality of life after surgery as the most important factor and daycase surgery as the least important. These results should be considered when planning future surgical trials and clinical guidelines.

**Electronic supplementary material:**

The online version of this article (doi:10.1007/s00268-016-3854-x) contains supplementary material, which is available to authorized users.

## Introduction

Ten to 15% of the Western population have gallstones, and approximately 70,000 cholecystectomies performed every year in the UK [1, 2]. In 2013, the Royal College of Surgeons of England (RCS) and the Association of Upper Gastrointestinal Surgeons (AUGIS) produced a commissioning guide for gallstone disease to enable clinical commissioning groups to “…. start a conversation with providers who appear to be ‘outliers’ from the indicators of quality that have been selected” [2]. These indicators of quality include items such as “Average Length of Stay”, “30-Day Readmission Rate” and “Daycase Rate”.

Such traditional outcomes are the focus of much ongoing research. Over a two-month period in 2014, hospitals across Great Britain were asked to enter data into the CholeS study which collected information on length of hospital stay, readmission rates and other factors such as length of procedure and degree of difficulty for the surgeon [3]. And a number of recent clinical trials have been powered to detect differences in post-operative pain scores [4], duration of surgery [5] and length of stay [6]. However, it is unclear how important these outcomes actually are to patients.

The aim of this study was to establish which factors were most important to patients admitted as an emergency with gallstone pathology. This was done using a survey produced by combining a list of outcomes from recent clinical trials with the opinions of patients. As a secondary analysis, surgeons and managers were asked to complete the same survey.

## Methods

### Development of patient survey

Three methods were used to develop the patient survey. First, a systematic review of the literature was performed (see supplementary material, *S1*). Using the PubMed database, a five-year period was searched from November 2009–October 2014. The keywords “gallstones” and “surgery” were used together with the “clinical trial” and “English language” filters. Initially, 67 studies were identified. This reduced down to 33 clinical trials after screening. In the 33 studies, 46 different outcomes were reported. After excluding duplicates (for example, post-operative pain was captured in the trials as post-operative pain scores on visual analogue scale 8, 24 h and 7 days, pain scores at 1, 6 h and 1 week, analgesics doses during the first 24 h and post-operative shoulder tip pain), a 30-item list of outcomes was taken forward into the patient survey.

Second, a pilot patient survey was performed to supplement the list of outcomes identified by the literature review. Ten patients about to undergo either urgent or elective cholecystectomy were given a blank sheet of paper and asked to write down the five factors most important to them at that time. An example of one of these “top 5 lists” is shown in the supplementary material, *S2*. Through this process, five additional factors of potential importance were identified: (1) nursing care; (2) cleanliness of the ward environment; (3) return to normal diet; (4) communication skills of the surgeon and (5) contact details post-procedure.

Third, additions to the survey were made based upon factors the investigators felt may be important but were not identified using the first two methods. These six items were: (1) staying under the care of the same consultant; (2) reputation of the consultant; (3) having surgery at the University hospital of South Manchester (UHSM); (4) UHSM’s ranking in national NHS surveys; (5) Opinions of friends and family about UHSM and (6) Stories about UHSM in the local/national press.

### Conduction of patient survey

A 41-item survey was brought forward to the main study, which was conducted over an eight-month period from November 2014–June 2015. Research and development approval was obtained locally from the UHSM R&D department. Patients admitted as an emergency to the Surgical Admissions Unit (SAU) at UHSM with gallstone pathology were surveyed prospectively, after obtaining verbal consent. They were approached after a diagnosis of gallstone pathology had been made on imaging, but before either surgery was performed or they were discharged and given a date for early elective surgery. They were given an explanation about the purpose of the study and were then asked to read a standardised patient information sheet about laparoscopic cholecystectomy before completing the survey. Next to each survey item was a 10-cm-long visual analogue scale. Patients were asked score each item using this scale; thus, a mark 7.6 cm along the scale corresponded to a score of 76/100. A copy of this survey is shown in the supplementary material, *S3*.

### Inclusion and exclusion criteria

All adult patients admitted to the SAU with biliary colic, cholecystitis, predicted mild pancreatitis, common bile duct stones and cholangitis were included. Patients who were unwell with pancreatitis and thus not suitable for urgent cholecystectomy and those deemed not fit for surgery due to comorbidities were excluded. Patients unable to read and write and those that could not make informed decisions about their own treatment were also excluded.

### Secondary analyses

The same survey was given to surgeons and hospital managers. All were blinded to the results of the patient survey. The surgeons were ST3 (resident) level and above. The managers included staff from the surgical directorate, waiting list office and the surgical ward managers.

### Statistical analyses

The survey data were left-skewed; therefore, results are expressed as median (range). All analyses were performed using STATA version 13.1 (College Station, Tx, USA).

## Results

### Patient cohort

Of the 57 patients who were approached, 56 completed the survey. Forty-three were female; median age 51 years (range 21–82). Diagnoses of the surveyed patients, in descending order of frequency, were: cholecystitis (28 patients), biliary colic (13), pancreatitis (10), common bile duct stones (3) and cholangitis (2).

### Patient survey

**Fig. 1:**
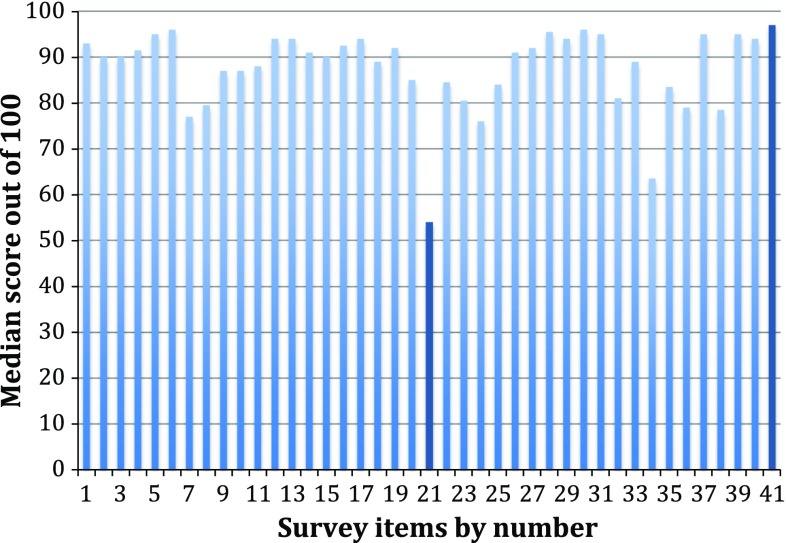
Median patient cholecystectomy survey scores (*n* = 56) with the highest- and lowest-ranking items shown in *dark blue*

These results are displayed in Fig. 1. The top-scoring survey item was “long-term quality of life after surgery”, with a median value of 97 out of 100. Other top-scoring items included “cleanliness of the ward environment” and “pain control after surgery” (both 96), “communication skills of the surgeon” (95.5) and “nursing care”, “having surgery at UHSM”, “risk of ongoing pain after gallbladder surgery” and “overall patient satisfaction” (all 95).

The lowest-scoring item was “being treated as a daycase” (median 54). Other items ranked in the bottom five were “stories about UHSM in the local/national press” (63.5), “short time to return to normal diet” (76), “operative duration” (77) and “cosmetic outcome” (78.5).

To test the internal validity, survey responses were divided up into two time periods and compared: November 2014–February 2015 (*n* = 26) and March 2015–June 2015 (*n* = 30). Quality of life was the top-scoring outcome in the first time period and was ranked second behind nursing care in the second; daycase surgery was the lowest-ranked in both time periods. This showed the results were consistent over time.

### Surgeon survey

**Fig. 2:**
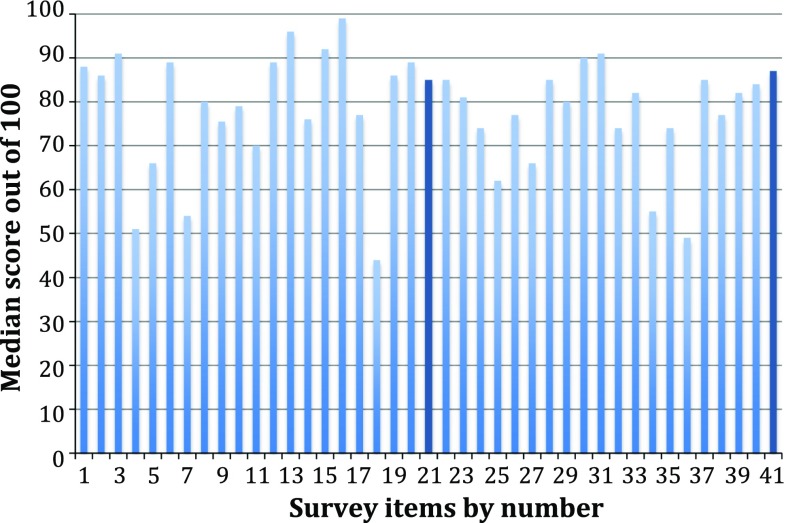
Median surgeon cholecystectomy survey scores (*n* = 13) with the items ranked highest and lowest by patients shown in *dark blue*

These results are displayed in Fig. 2. Thirteen surgeons completed the survey (five consultants and eight ST3 + doctors). Overall, the surgeons gave lower median scores than the patients. The item ranked highest by surgeons was “risk of bile duct injury” (99). Other high-ranking outcomes included “severe post-operative complications” (96), bile leak (92), low numbers of hospital visits and standards of nursing care (both 91). The lowest-ranking item was post-operative liver enzyme levels (44). The median score for long-term quality of life was 87, equating to a rank of 11 out of 41.

### Manager survey

**Fig. 3:**
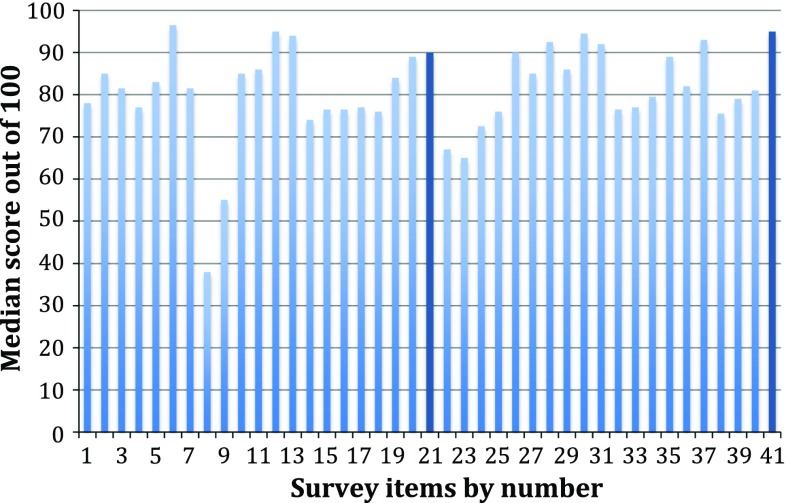
Median manager cholecystectomy survey scores (*n* = 8) with the items ranked highest and lowest by patients shown in *dark blue*

These results are displayed in Fig. 3. Eight managers completed the survey. The highest-ranking outcomes were “post-operative pain control” (96.5), “long-term quality of life after surgery” and “overall risk of complications” (both 95), “cleanliness of the ward environment” (94.5) and “risk of severe complications” (94). The lowest-ranking factor was conversion to open surgery. Cost was ranked 33rd highest by the managers.

## Discussion

### Key findings

Long-term quality of life after surgery is the most important factor for patients requiring a cholecystectomy following an emergency presentation with gallstone pathology. Other factors of importance include pain control, cleanliness of the ward environment and communication skills of the surgeon. Daycase surgery was the item ranked lowest by the patients, but it is a key measure of quality in the 2013 AUGIS and RCSEng Gallstone Disease Commissioning Guide [2]. Other low-ranking items included operative duration, cosmetic outcome and conversion from open surgery. Surgeons regard post-operative complications as most important—risk of bile duct injury, bile leak and major complications were all in the surgeons’ top-five. They regard long-term quality of life as important, but only ranked it 11th highest.

### Comparison with published literature

Hey et al. [7] showed patients photographs of scars after standard cholecystectomies versus single-incision (SILS) procedures and presented outcome data as well, with the majority of patients (86%) preferring the standard technique. In a similar study, Dauser et al. [8] asked patients to rank outcomes in order to compare the efficacy of single-incision and conventional cholecystectomies. Patients rated risk of complications and a surgeon’s experience as more important than cosmesis and length of stay.

Results of post-operative patient satisfaction surveys are inconsistent. One Dutch study found around 90% of patients considered their outcome to be good [9], whereas in a larger Finnish study, more than one-third of patients experienced persistent abdominal symptoms after surgery [10].

There have been numerous clinical trials evaluating surgical techniques and technologies during laparoscopic cholecystectomy, and many of these have been reviewed by the Cochrane hepatobiliary group. They frequently identify the lack of data on quality of life and time to return to normal activities within these studies and have recommended that these factors be introduced into future trial designs [11, 12].

Surgeons appear to be taking heed of this. In a recent randomised controlled trial comparing cholecystectomy and intra-operative cholangiogram with endoscopic duct assessment followed by cholecystectomy from the group in Geneva, quality of life was assessed as a secondary endpoint using EuroQol-5D scores [6]. And, quality of life was a secondary endpoint in another recent Swiss study evaluating cosmesis and body image after SILS versus conventional laparoscopic cholecystectomy [13].

### Strengths and limitations

This study has several strengths. First, the 41-item survey was comprehensive and reflected the views of patients and surgeons because it was produced using results from a systematic review of the literature—incorporating endpoints from clinical trials in the last five years—and augmented by a preliminary patient questionnaire. Second, patients were surveyed prospectively before surgery, after being informed they require a cholecystectomy and had read an information leaflet. Thus, responses were contemporaneous and obtained from well-informed individuals, meaning surgeons can apply these findings to this group of patients in their own practice. Finally, this was a novel study that produced surprising results and highlighted differences between what surgeons and national policy makers perceive to be of importance and the opinions of patients themselves.

A limitation of this study centres on potential differences in baseline knowledge of the three groups completing the survey. It can be argued that surgeons scored “risk of bile duct injury” highest because they know it can have a devastating effect upon patients’ quality of life, whereas “long-term quality of life” is a broader term that includes other factors they may have perceived to be of less importance (such as time to return to normal activities, body image and recurrent symptoms). Therefore, if patients completing this survey had a more in-depth knowledge about the consequence of bile duct injuries, they may have given this a higher score. A further weakness may relate to the way questions were perceived by patients at that point in time. It is possible that individuals who were unwell on SAU will have struggled to relate to “daycase surgery”. And those who had experienced symptoms for a long time prior to their emergency admission may have assumed (sometimes mistakenly) that the cholecystectomy would improve their quality of life and as a result, other aspects of their general health. Finally, the explicit inclusion and exclusion criteria mean these findings can only be applied to this specific group of patients, which affects their generalisability. It is unclear what priorities other groups of patients have, for example, those who are unfit for cholecystectomy, or patients seen in the outpatient clinic who are being counselled about an elective cholecystectomy.

### Clinical implications

Gallstones typically affect women of working age. Clearly, from the results of this study, they have a big impact on quality of life and as surgeons we must consider ways in which to improve patients’ hospital experience and convalescence. Given that peri-operative pain control and communication skills were also rated highly, these are areas where potential improvements should be focused. There is a tendency for surgeons to expend efforts on improving the surgery—making it easier, making it faster, getting patients out of hospital sooner. But maybe we should take a step back and consider what patients want and when we design future studies to evaluate novel surgical techniques, power them to detect improvements in quality of life rather than traditional “surgical” outcomes such as operative duration or cosmesis score.

## Conclusion

This study highlights the disconnect that may exists between the opinions of surgeons and patients, in this case with regard to gallstone pathology. There is substantial momentum behind further research and data collection on operative duration, conversion rates and daycase surgery for gallstones but very little about patient satisfaction and quality of life. Gallstones affect young people of working age and they have a big impact on their daily lives and future studies and national guidelines should take account of this.

## Electronic supplementary material

Below is the link to the electronic supplementary material.

Supplementary material 1 (DOCX 2225 kb)
