# Supplementary material for: Patient-Reported Outcomes for Acute Gallstone Pathology
Source: World J Surg. 2017 Jan 10;41(5):1234–8. doi: 10.1007/s00268-016-3854-x (PMC5394152; doi:10.1007/s00268-016-3854-x)
Supplement: Supplementary file 1 — Supplementary material 1 (DOCX 2225 kb) [file 268_2016_3854_MOESM1_ESM.docx]

Citations in PubMed November 2009 – October 2014

N = 67

*S1* PRISMA Flow diagram showing studies identified by the systematic review

Full-text articles assessed for eligibility

N = 35

Records excluded based on title / abstract

N = 32

Abstracts of clinical trials in gallstone surgery

N = 33

Records excluded based on full-text article

N = 2


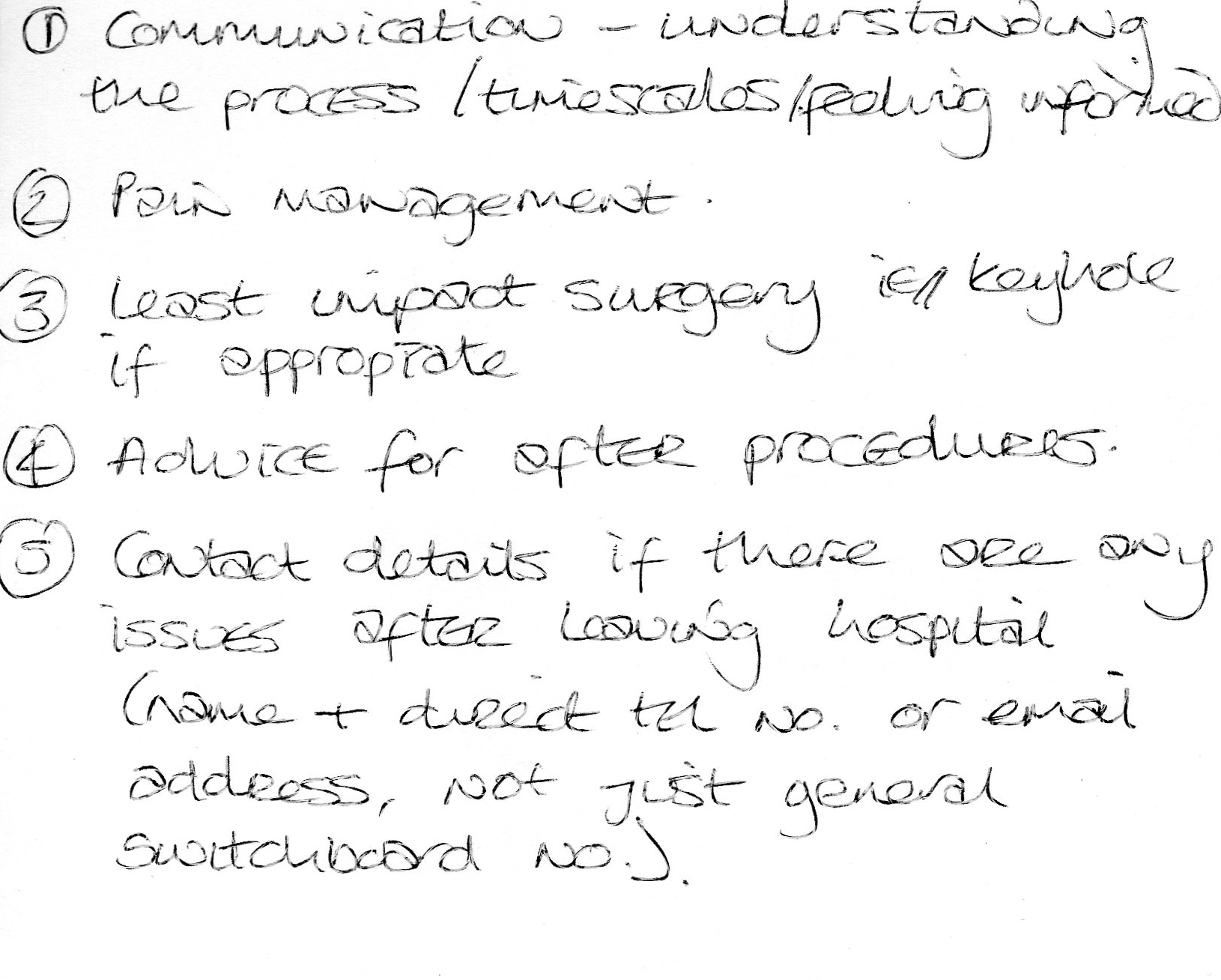


*S2* Photograph of typical patient responses in the pilot survey

***What outcomes are important?***

You have been admitted with gallstones and need surgery to remove your gallbladder. We are interested in finding out which factors/outcomes patients with gallstones view as high priority.

Below is a list of potential outcomes from gallbladder surgery. Some of these outcomes may not be as important or relevant from a patient’s point of view, but might be important to healthcare professionals involved in your care.

Using the 1-100 scale, please score each outcome by drawing a vertical line across, as shown in the example below (a line drawn at 100 being of maximum importance).

1

100

**Logistical issues**

100

1. Having the gallstones treated/removed on the same admission

1

1

1

1

1

100

1. Short waiting time to gallbladder surgery
2. Low number of hospital visits/appointments

100

100

1. Staying under the care of the same consultant

100

1. Having the surgery here at Wythenshawe

**Surgical outcomes**

1

1

1

1

1

1

100

1. Pain control before and after the surgery

100

1. Short operative duration (mins)
2. Conversion from keyhole to open surgery

100

100

1. Difficulty of surgery for the surgeon
2. Amount of blood lost during the surgery

100

1. Spillage of bile during the operation

100

**Post-operative complications**

1

1

1

1

1

1

1

1

100

1. Overall risk of complications following the surgery

100

1. Risk of severe post-operative complications (e.g. return to theatre)

100

1. Risk of minor post-operative complications (e.g. wound infection)

100

1. Risk of bile leak

100

1. Risk of bile duct injury

100

1. Risk of retained (missed) gallstones
2. Post-operative liver enzyme levels (blood tests)

100

1. Risk of readmission with gallstones

100

**Recovery**

1

1

1

1

1

100

1. Short length of stay in hospital (days)

100

1. Being treated as a day case (no overnight stay)

100

1. Short time taken to return to normal level of function (e.g. driving)

100

1. Short time taken off sick before returning to work
2. Short time before returning to a normal diet

100

**Cost**

100

1

1. Cost (£) of the hospital stay/procedure to the NHS

**The hospital experience**

1

1

1

1

1

1

1

1

1

100

1. Patient involvement in decisions about treatment (e.g. timing of surgery)

100

1. Dietary advice (before and after the surgery)

100

1. Communication skills of the surgeon
2. Reputation of the consultant surgeon and their team

100

1. Cleanliness of the ward/theatre area

100

100

1. Standards of nursing care

100

1. Standards of hospital food

100

1. Contact details for the consultant/ward after discharge home
2. Stories about Wythenshawe hospital in the local/national press

100

1. Opinions of friends and family about Wythenshawe

1

1

100

1. Wythenshawe hospital’s ranking in national NHS surveys

100

**Long-term outcomes**

1

100

1. Overall patient satisfaction with the procedure

1

100

1. Cosmetic outcome (appearance of the surgical scar)

1

100

1. Residual abdominal symptoms (ongoing pain after gallbladder removal)

1

100

1. Risk of long-term complications related to the surgery (e.g. hernia)

1

100

1. Long-term quality of life after surgery

**Other outcomes**

Please use this section to document (and score out of 100) any other outcomes not listed above that you feel are important

1

1

1

100

__________________________________________________________

100

100

__________________________________________________________

__________________________________________________________
